# A case report and review of literature: Tuberculous pericarditis with pericardial effusion as the only clinical manifestation

**DOI:** 10.3389/fcvm.2022.1020672

**Published:** 2022-11-02

**Authors:** Shipeng Wang, Jingyue Wang, Junqian Liu, Zhiyu Zhang, Jiahuan He, Yushi Wang

**Affiliations:** Department of Cardiology, The First Hospital of Jilin University, Changchun, China

**Keywords:** tuberculosis, tuberculous pericarditis (TBP), pericardial effusion (PE), diagnosis, case, review

## Abstract

Tuberculosis is a main cause of pericardial disease in developing countries. However, in patients with atypical clinical presentation, it can lead to misdiagnosis, missed diagnosis, and delayed treatment. In this study, we report a case of a 61-year-old woman admitted to the cardiac intensive care unit with “weakness and loss of appetite” and a large pericardial effusion shown by echocardiography. After hospitalization, a pericardiocentesis was performed, and the pericardial fluid was hemorrhagic. However, the Xpert MTB/RIF and T-SPOT tests were negative, and repeated phlegm antacid smears and culture of pericardial fluid did not reveal antacid bacilli. The patient eventually underwent thoracoscopic pericardial biopsy, which revealed extensive inflammatory cells and significant granulomas. Combined with the fact that the patient’s pericardial effusion was exudate, the patient was considered to be suspected of tuberculous pericarditis (TBP) and given empirical anti-tuberculosis treatment the patient’s symptoms improved and the final diagnosis was TBP. In this case report, it is further shown that a negative laboratory test cannot exclude tuberculosis infection. In recurrent unexplained pericardial effusions, the pericardial biopsy is feasible. In countries with a high burden of tuberculosis, empirical antituberculosis therapy may be used to treat the pericardial effusion that excludes other possible factors.

## Introduction

Tuberculosis (TB) is one of the top 10 leading causes of death worldwide and the leading cause of death by a single source of infection. According to the World Health Organization (WHO) global TB report 2021 version (1), China had the second highest incidence of TB in the world in 2020, accounting for 8.5% of TB globally. While extrapulmonary TB accounts for 15% of all TB cases, an epidemiological study indicated that nearly one-third of HIV-negative TB patients in China have extrapulmonary TB (2). TB is caused by mycobacterium tuberculosis and is mainly transmitted through the respiratory system. Mycobacterium tuberculosis mainly affects the lungs and causes pulmonary tuberculosis, but the rest of the body can also be infected. The clinical presentation of tuberculosis is variable, and symptoms are often atypical, which makes it more challenging to diagnose early. Tuberculous pericarditis (TBP) is a rarely seen form of extrapulmonary TB, accounting for 1–2% of all TB infections. TBP is the most common cause of massive pericardial effusion in developing countries. It is also the most common cause of constrictive pericarditis in adults, which has a poor prognosis and high mortality rate owing to constrictive pericarditis (3). Therefore, early diagnosis and intervention of TBP are crucial to treat the disease and improve the prognosis. In this article, we describe a patient with TBP who was admitted to the cardiovascular intensive care unit with massive pericardial effusion, her clinical presentation, diagnostic process, and treatment. We share our experience in the diagnosis of TBP.

## Case presentation

A 61-year-old Chinese woman presented with weakness and loss of appetite for the previous 20 days. She had a medical history of cholecystitis and underwent a cholecystectomy several years ago. Physical examination showed that the patient had a temperature of 36.7°C, blood pressure of 128/76 mmHg, and a heart rate of 77 beats/min. There were coarse breath sounds in both lungs and diminished breath sounds in both lower lungs; a small number of wet rales could be heard, and diminished heart sounds with enlarged heart borders due to pericardial effusion.

**FIGURE 1:**
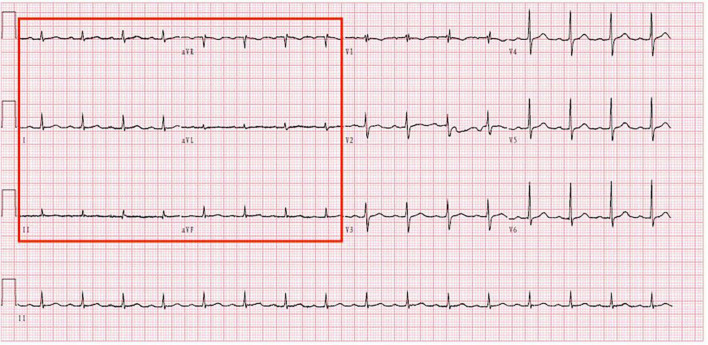
Electrocardiogram shows that the patient has low voltage in the limb leads.

**FIGURE 2:**
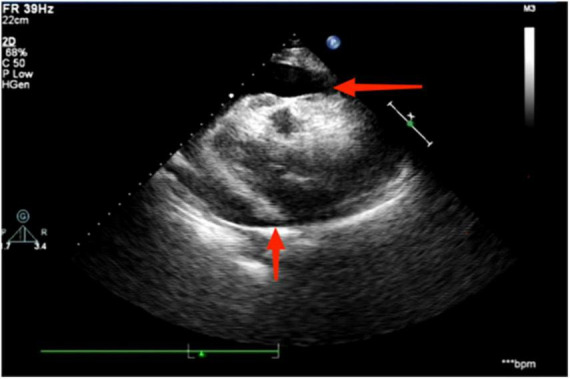
Echocardiography suggests the presence of a moderate to large pericardial effusion, as indicated by the red arrowhead.

**FIGURE 3:**
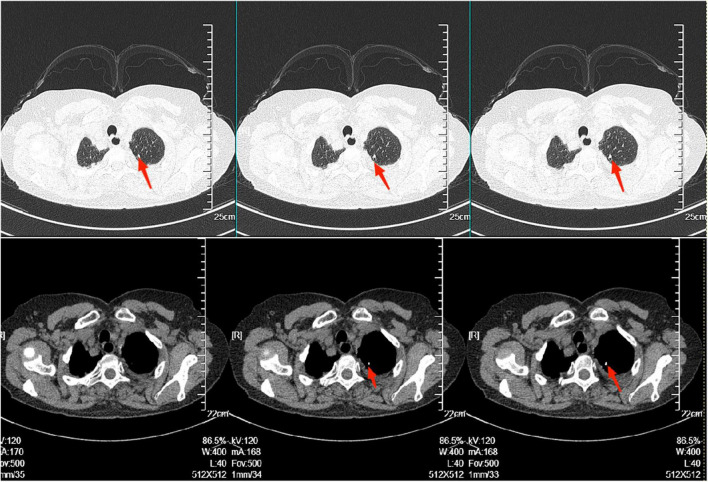
There was scattered inflammation in both lungs, inflammatory nodules in the upper left and lower right lobes. Calcification in the left upper lobe, as shown in the red arrow in the figure. The radiologist considered it to be stable tuberculosis.

The electrocardiogram (Figure 1) suggested a sinus heart rate with low voltage in the limb leads. The patient’s laboratory data at admission are shown in Table 1. Echocardiography indicated moderate to severe pericardial effusion (Figure 2), and echo-free areas were seen in the pericardial cavity. The diastolic fluid width was measured as 14 mm in the anterior wall of the right ventricle, 16 mm in the posterior wall of the left ventricle, 19 mm in the lateral wall of the left ventricle, and 20 mm in the apical part of the left ventricle. A bedside color doppler ultrasound showed bilateral pleural effusions. After admission, the patient had a fever intermittently, with a temperature of up to 38.3°C without chills and shivering. The patient’s temperature was elevated within 24 h of admission, meaning that the possibility of community-acquired pneumonia could not be excluded. After blood cultures were retained, the patient was also given empirical anti-infective treatment with moxifloxacin. However, as the patient’s improvement in relevant tests was considered not to exclude tuberculosis, the use of fluoroquinolones alone should be avoided, and the patient’s moxifloxacin was discontinued and changed to ampicillin. The patient’s blood culture later indicated no bacterial growth. A computed tomography (CT) scan of the lungs revealed sporadic inflammation in both lungs, inflammatory nodules in the upper left and lower right lung lobes, calcification in the upper left lobe (Figure 3), and bilateral pleural effusions. Furthermore, the mediastinal lymph nodes were enlarged and partially calcified. Pericardiocentesis and drainage were used to relieve the patient’s symptoms, such as chest tightness and shortness of breath. The patient’s pericardial effusion was hematogenous, with routine results as follows: protein 53.96 g/L; Reye’s test positive; total erythrocyte count 214300.00 × 10^6^/L; total leukocyte count 3762.00 × 10^6^/L; percentage of single nucleated cells 84%; adenosine deaminase (ADA) 32.0 U/L; lactate dehydrogenase (LDH) 326 U/L; and carcinoembryonic antigen (CEA) 0.47 ng/ml.

**TABLE 1:** The patient’s laboratory data.

Parameter	Values		Unit
	
	Day 1	References value	
WBC	6.06	3.5–9.5	10^9/L
NE#	4.53	1.80–6.30	10^9/L
NE	0.75	0.4–0.75	%
LY#	0.96	1.1–3.2	10^9/L
LY	0.16	0.2–0.5	%
Hb	82	115–150	g/L
PLT	342	125–350	10^9/L
AST	25.5	13.0–35.0	U/L
ALT	47.3	7.0–40.0	U/L
TB	8.6	0.0–21.0	μmol/L
DBIL	2.3	0.0–6.8	μmol/L
IBIL	6.3	5.0–20.0	μmol/L
Lac	0.6	0.5–2.2	mmol/L
Fib	4.34	1.8–4.0	g/L
TP	60.3	68–85	g/L
GLU	5.8	3.9–6.1	mmol/l
BUN	2.36	3.1–8.8	mmol/L
sCr	50.1	41–81	μmol/L
TG	0.88	0.28–1.8	mmol/L
D-dimer	> 5000	100–600	ng/ml
CKMB	< 1.0	0–4.3	ng/ml
Tn	< 0.05	0–0.05	ng/ml
PT	14.7	9.0–13.0	S
INR	1.24	0.8–1.2	–
APTT	24.5	21–33	S
PCT	< 0.05	0–0.5	ng/mL
CPR	54.23	0–3.5	mg/L
TSH	1.781	0.35–4.94	μIU/ml
FT3	2.31	2.43–6.01	pmol/L
FT4	12.43	9.01–19.05	pmol/L

WBC, white blood cell; NE, neutrophils; LY, lymphocyte; Hb, hemoglobin; PLT, platelets; AST, aspartate aminotransferase; ALT, alanine transaminase; TB, total bilirubin; DBIL, direct bilirubin; IBIL, indirect bilirubin; Lac, lactic acid; Fib, fibrinogen; TP, Total proteins; GLU, glucose; BUN, blood urea nitrogen; sCr, serum creatinine; TG, triglyceride; CKMB, creatine kinase MB; Tn, troponin; PT, prothrombin time; INR, international normalized ratio; APTT, activated partial thromboplastin time; PCT, procalcitonin; CRP, C-reactive protein; TSH, thyroid-stimulating hormone; FT3, free triiodothyronine; FT4, free thyroxine.

The above results indicated that the patient’s pericardial effusion was exudate. The pericardial effusion smear did not find acid-fast bacilli, and Xpert MTB/RIF (Xpert Mycobacterium TB/RIF test is a new test, which is helpful for rapid diagnosis of TB and drug resistance, thus bringing revolutionary changes to TB control.) and T-SPOT tests (The T-SPOT test is a unique TB blood test designed to reduce variability and maximize sensitivity, even in people with low immune function.) were negative. The exfoliated cells of the pericardial effusion showed lymphocytes and lobulated nuclei, and no cancer cells were found. Female tumor markers such as CEA, cancer antigen (CA) 125, CA 199, and neuron-specific enolase were below the reference values to exclude tumor-derived pericardial effusion. Further positron emission tomography/computed tomography (PET/CT) was performed, which showed inhomogeneous thickening of the pericardium, enhanced metabolism, mediastinal lymph nodes, and uterine metabolism fibroids. Therefore, we could almost exclude the possibility that pericardial effusion was caused by a tumor. The patient’s thyroid function test suggested that her free triiodothyronine (FT3) of 2.31 pmol/L was only mildly depressed, and it was unlikely that the pericardial effusion was caused by hypothyroidism. To rule out the possibility that the patient’s pericardial effusion was caused by autoimmune disease, we further performed autoimmune marker screening, and the patient’s antinuclear antibodies (ANA), anti-neutrophil cytoplasmic antibodies (ANCA), and antiphospholipid. The antiphospholipid syndrome antibodies were all negative, which essentially excluded autoimmune diseases.

Three days after admission, the patient had no fluid flow from the pericardial drainage tube, so the pericardial drainage tube was removed. Seven days after admission, the patient’s bedside color doppler ultrasound showed that the right pleural effusion was less than at the time of admission. However, there was still a considerable amount of pleural effusion on the left side. We gave the patient left-sided thoracic puncture drainage, and the drainage fluid was light-red. Routine examination of the pleural fluid showed protein 42.83 g/L; a positive Reye’s test; total red blood cell count of 67,500.00 × 10^6^/L; total white blood cell count of 1359.00 × 10^6^/L; and percentage of single nucleated cells 97%. Its property was approximately the same as that of pericardial effusion, which was an exudate and the possibility of extravasation of a large amount of pericardial effusion could not be excluded. After receiving symptomatic supportive treatment, the patient’s symptoms showed improvement, and the family refused to undertake further specific investigations to determine the cause.

**FIGURE 4:**
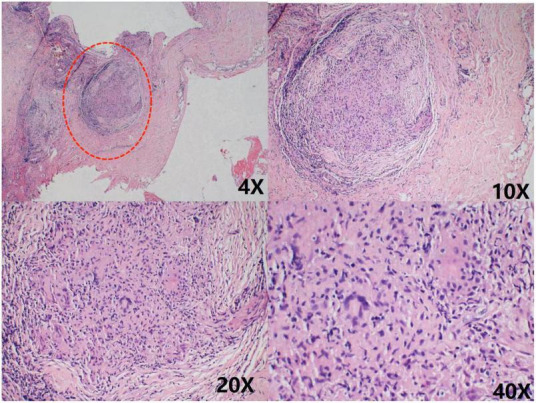
Chronic inflammatory cell infiltration was seen in the connective tissue granulomatous lesions and fibrous tissue hyperplasia were seen in the lymph nodes No necrosis was observed. As shown in the red circle.

**FIGURE 5:**
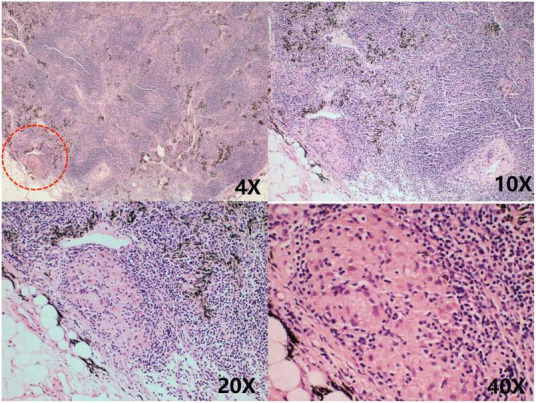
Granulomatous lesions are seen within the fibrofatty tissue, as shown in the red circle.

However, 2 months later, the patient was readmitted with malaise, chest pain, and low-grade fever. She had been treated with cephalosporin at a local hospital before admission, but the outcome was poor. A small amount of pericardial effusion was again detected by echocardiography at the time of admission. To determine the cause of the pericardial effusion, the patient was referred to the department of thoracic surgery for a biopsy of the pericardial mass. Pathological findings showed granulomatous lesions and fibrous hyperplasia of the mediastinal lymph nodes (Figure 4) and granulomatous lesions of the pericardial fibrofatty tissue (Figure 5). Furthermore, the patient’s pulmonary CT indicated a nodular calcified shadow in the left upper lung and enlarged and partially calcified lymph nodes in the mediastinum and part of the bilateral hilum. The patient was considered to have stable tuberculosis in the upper lobe of the left lung, and the pericardial puncture fluid was exudate with increased leukocytes, mainly monocytes, and mildly elevated ADA. TBP can be suspected according to the diagnostic criteria, and the patient was advised to go to an infectious disease hospital for further treatment. The dosing regimen was: isoniazid 300 mg orally one time a day (QD), rifampicin 600 mg orally QD, ethambutol 100 mg orally QD, and pyrazinamide 100 mg orally QD. The four drugs were administered for 8 weeks and then reduced to isoniazid and rifampicin to continue the treatment for 6 months. According to the PET-CT results, the patient’s pericardium had become unevenly thickened. To avoid progression to constrictive pericarditis, the patient was given prednisolone 50 mg, and the dose was gradually reduced. After 8 months, we followed up with the patient by telephone; her symptoms, such as weakness and chest pain, were improved following the anti-tuberculosis treatment.

## Discussion

We have reported the case of a patient who was diagnosed with TBP by pericardial biopsy after all laboratory tests were negative. The patient presented with fatigue and loss of appetite as the primary clinical manifestations, and an echocardiogram showed moderate to large pericardial effusion. The drainage fluid after the puncture was a hemorrhagic exudate. Tuberculosis and cancer are common causes of pericardial effusion, and TB is the most frequent cause in developing countries (4).

Extrapulmonary tuberculosis (EPTB) can affect any part of the body; it is often missed or misdiagnosed because clinical manifestations may be atypical, diagnostic tissue samples are readily unavailable, and bacterial levels are low, making diagnosis difficult (5). Pleura and lymph nodes are the most common sites of EPTB lesions, followed by bones, joints, intestines, peritoneum, kidneys, the genitourinary system, and meninges (6). A study on the epidemiology of extrapulmonary TB in Chinese inpatients suggested that skeletal TB may be the predominant form of extrapulmonary TB in China, accounting for nearly 41% of its EPTB; this discrepancy may be related to BCG vaccination nationwide in China (2). In contrast, pericardial TB is a rare type of EPTB, usually caused by retrograde dissemination of mycobacterium tuberculosis from the peripheral trachea, peripheral bronchi, or mediastinal lymph nodes *via* lymphatics or hematogenous dissemination of the primary TB infection (7). EPTB can present different physical signs and symptoms depending on the organ involved, the disease’s aggressiveness, and the host’s immune response. TBP can be divided into four stages, with corresponding clinical manifestations (8, 9). The mortality rate of TBP 6 months after diagnosis is 17–40% (10). Therefore, an early and effective diagnosis of TBP can help improve the disease cure rate and reduce mortality.

To facilitate diagnosis in TB-endemic countries, there are uniform diagnostic criteria for confirmed and suspected TBP based on clinical and laboratory findings (11). Currently, the most widely used laboratory tests for diagnosing TBP include T-SPOT, Xpert MTB/RIF, ADA, etc. The overall sensitivity and specificity of T-SPOT are 91 and 88%, respectively (12), while the corresponding figures for Xpert MTB/RIF are 68 and 99% (13). Hence, the latter is more specific than T-SPOT but has lower sensitivity when negative. The threshold value of ADA remains controversial; ≥ 40 U/L is usual when diagnosing the possible presence of TBP in patients, and the sensitivity and specificity of the test are 84.0 and 80.0%, respectively, at the threshold value of 40 U/L (14). Pericardial fluid culture is also a widely used test for diagnosing TBP; however, it is usually not preferred because it is time-consuming, and in 27–48% of cases, no tuberculous fraction is found in the pericardial fluid (15). Notably, this patient differed from typical patients with tuberculous cystitis in having no typical TB symptoms. She also recorded negative T-SPOT and Xpert MTB/RIF tests, ADA of 32 U/L in the pericardial fluid, and negative results for the culture of antacid bacilli in the pericardial fluid, which do not form the basis for a TBP diagnosis. A meta-analysis found the overall sensitivity and specificity of interferon-gamma (IFN-γ) to be 97 and 99%, respectively. However, IFN-γ is relatively expensive and is not commonly used as a laboratory test (16).

The patient was discharged with symptomatic treatment, but she was readmitted 1 month later with malaise, chest pain with hypothermia, and echocardiography indicated a small pericardial effusion. A single-center study involving 174 patients suggested that pericardial tissue biopsy is feasible for recurrent pericardial effusions to improve the likelihood of diagnosis in patients (17). Ultimately, this patient was diagnosed with TBP when a pericardial biopsy was performed to identify the source of the pericardial effusion, and a granuloma was found.

The current treatment for TB pericarditis is a “quadruple” regimen of rifampin, isoniazid, pyrazinamide, and ethambutol for 2 months, followed by isoniazid and rifampin for 4 months (3). Adjuvant corticosteroid therapy reduces the hospitalization rate for TBP and the incidence of patients progressing to constrictive pericarditis, but it increases cancer incidence in HIV-positive patients (18). Meanwhile, some studies have suggested that routine pericardiocentesis with prolonged drainage reduces the incidence of constrictive pericarditis (19, 20). In countries with a high burden of tuberculosis, ATT may be used to treat the pericardial effusion that excludes other possible factors (21). However, the efficacy and safety of empirical ATT need further study.

Our study also has a few limitations. The diagnosis of EPTB is a challenge. We have no definitive diagnostic evidence - mycobacterium tuberculosis was found or pericardial biopsy found caseous granuloma. There is still much room for exploration in the way of laboratory test of extra-pulmonary tuberculosis.

In conclusion, negative routine laboratory tests for the pericardial effusion of unknown origin does not exclude tuberculosis pericarditis. And a biopsy of the pericardial effusion may help clarify the diagnosis based on excluding other causes. Empirical anti-TB treatment may be considered after non-TB causes have been ruled out to the extent possible.

## Data availability statement

The original contributions presented in this study are included in the article/supplementary material, further inquiries can be directed to the corresponding author.

## Author contributions

SW conceived the idea and conceptualized the case. JW, JL, ZZ, and JH collected the data. SW analyzed the data and drafted the manuscript, which was then reviewed by YW. All authors contributed to the article and approved the submitted version.
